# RepD3D: A tool for representative period identification and associated boundary condition extraction

**DOI:** 10.1016/j.mex.2024.103109

**Published:** 2024-12-13

**Authors:** Clayton Cyril Soares, Arne Knies, Christian Winter

**Affiliations:** Institute of Geosciences, University of Kiel, Germany

**Keywords:** GUI, Representative period, Boundary conditions, Numerical model, Delft3D, EasyGSH, COSMO, Extraction, Hydrodynamics, RepD3D – support toolbox for modelling studies

## Abstract

Defining representative periods is a critical step for numerical modelers seeking to understand regional hydro- and morphodynamics in situations when long-term simulations are not feasible or future boundary conditions are unknown. However, identifying these periods and integrating the associated boundary conditions can be a time-intensive process. To address this, RepD3D has been developed as a Windows-based user interface, accompanied by open-source Python code, which helps users formulate and support modeling studies by employing a wind-based unfiltered reduction technique to identify representative periods. This tool is particularly beneficial for modelers investigating shallow-water wind-wave-driven processes or the influence of wind in their area of interest. For studies focusing on extreme events or specific wind directionality, RepD3D offers selective-class correlation, enabling users to identify representative periods for specific wind speed and direction classes. This approach avoids synthetic simulations and preserves the natural state within a simulation. Additionally, the application supports boundary condition extraction for user-defined models and conversion into Delft3D-4-compatible input files. RepD3D integrates water level and wave data from EasyGSH (1996–2016, German North Sea) and wind data from COSMO-REA6 (1995–2019, Europe), with its open-source design allowing for adaptation to other datasets.

• RepD3D is a pre-compiled tool for Windows, designed to identify wind-based unfiltered and reduced representative periods and extract associated boundary conditions for modeling studies.
• A modified version of the all-class correlation method, known as selective-class correlation, is introduced to identify representative periods specific to user-desired wind speeds and directions.
• Boundary conditions for German North Sea (EasyGSH) and Europe (COSMO-REA6) are extracted and converted to DelftD3D-4-readable input files.

## Graphical abstract

**Figure:**
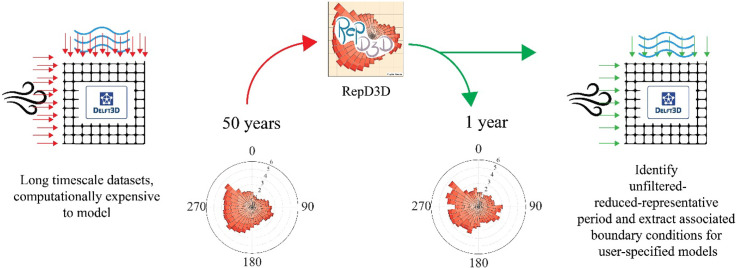

Specifications table

**Table:** 

Subject area:	Earth and Planetary Sciences
More specific subject area:	Coastal dynamics – Hydro/Morphodynamic modelling
Name of your method:	RepD3D – support toolbox for modelling studies
Name and reference of original method:	Soares, C.C., Galiforni-Silva, F., Winter, C., 2024. Representative residual transport pathways in a mixed-energy open tidal system. Journal of Sea Research 201, 102530. https://doi.org/10.1016/j.seares.2024.102530
Resource availability:	Source code: https://github.com/capt-clay10/RepD3D.git

## Background

Process-based numerical models are widely used to enhance the understanding of hydrodynamic, sediment transport and morphodynamic systems around the world, providing critical insights into complex environmental processes [8,9,12]. The performance of these models is inherently tied to the characteristics of the boundary conditions under which they are simulated. In many cases, the input conditions for simulations are selected based only on their availability rather than their representativeness to the system being studied. This approach can lead to misinterpretations, particularly when the goal is to achieve a representative understanding of the system dynamics.

Even when long-term input data are available, running decadal-scale simulations is computationally costly, which also promotes the use of reduction techniques. Reduction techniques condense or simplify the forcing parameters by filters. Filtered parameters refer to forcing parameters that have been processed or modified using statistical or analytical techniques (such as thresholding), to reduce variability, identify specific trends, or focus on dominant features while excluding less significant fluctuations [2,4,13,17]. These methods, however, are often susceptible to user bias [3] and foster non-representation of natural state [16,21]. In general, to obtain a representative understanding of a system without compromising on computational efficiency, it is best to run an unfiltered simulation which is also reduced.

To address this challenge, this paper introduces RepD3D, a stand-alone Windows-based graphical user interface (GUI) application and python-based open-source code. It (1) assists users in identifying a representative basis for their unfiltered and reduced modeling objectives and (2) offers support in extracting the associated input conditions tailored to their objectives for use in Delft3D-4, a widely-used process-based modeling suite [5,15]. Previously, Dissanayake et al. [6] used an algorithm to identify a Representative Wind Year (RWY) by calculating a long-term average wind profile from historical data and selecting the year with the smallest deviation from this profile, based on weighted averages of wind speed and direction probabilities. However, this approach does not account for constituent distributional differences of wind occurrences or correlations between wind speed and direction, potentially misrepresenting the reference period. In contrast, RepD3D uses the algorithm developed by Soares et al. [18] which evaluates distributional similarity, incorporates speed-direction correlations, and considers anomalies before calculating a representativity score. Moreover, this algorithm offers the possibility to identify shorter periods with selective-class correlation. This feature allows users to identify specific representative periods that correspond only to user-specified wind directions and wind speeds, resembling their behavior within a reference period. This helps users in studying the impact of special conditional cases without resorting to synthetic conditions, keeping the natural state intact.

The secondary functionality of RepD3D supports boundary data extraction and conversion to Delft3D-4 [5,14] readable input files. It is currently only adapted to the water level and wave parameters from EasyGSH [10,11] in the German North Sea and wind fields from COSMO-REA6 [1] in Europe (see Fig. 5). Similar to the functionality of the Delft dashboard [19] model builder, this function produces boundary condition time-series files for user defined open boundaries. This functionality reduces the user load and provides a convenient and fast way to generate boundary condition files without resorting to complex coding environments (when using the GUI). Together, these two functionalities of RepD3D can help the user formulate and execute a process-based model with a strong basis. It is noted that while development of the open-source code will continue, the code is available to be adapted to different input databases.

## Method details

**Fig. 1:**
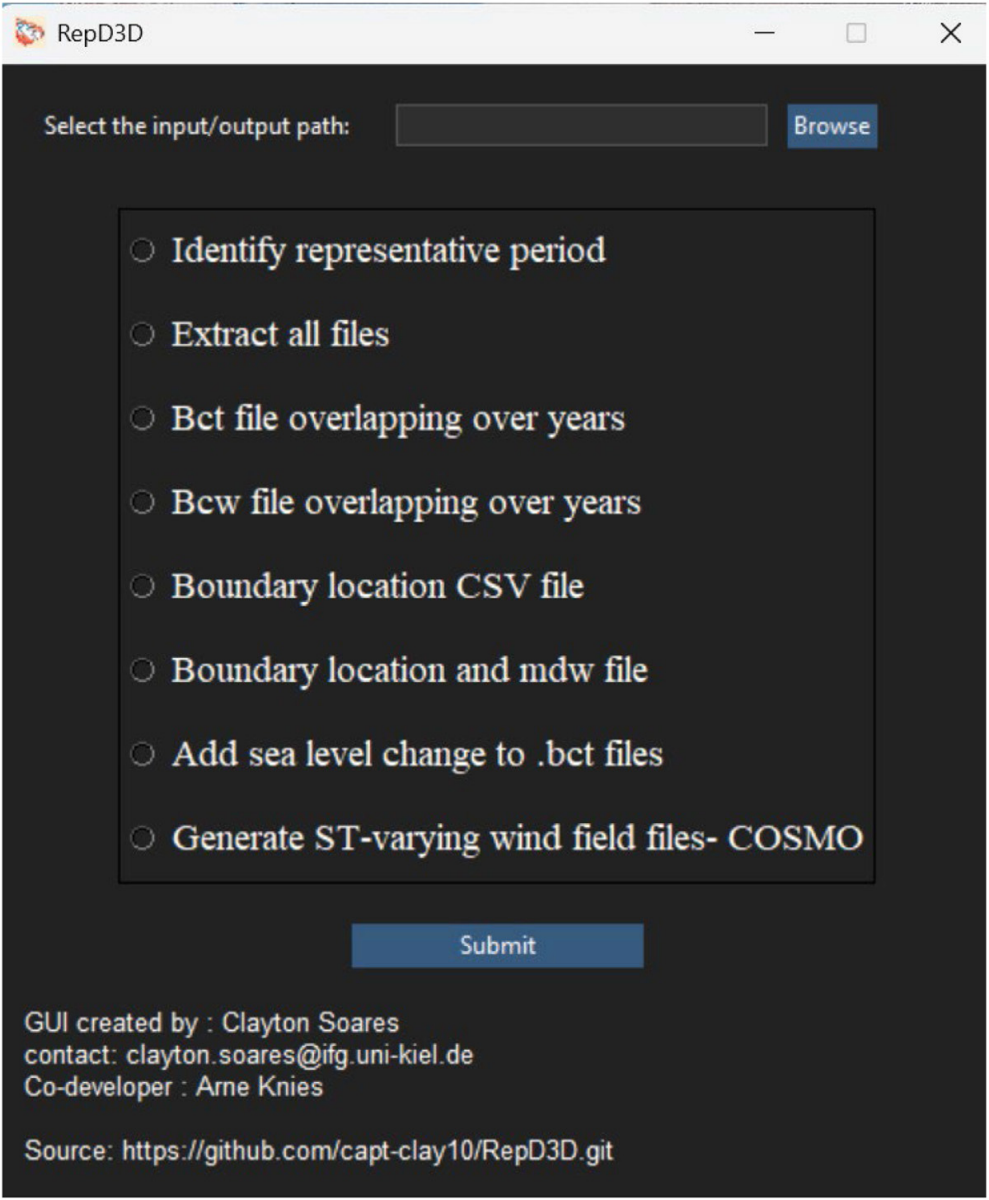
RepD3D main menu showing all the sub-modules.

RepD3D is available as source code and pre-compiled as a Windows 64 bit executable. The RepD3D executable file is a GUI application (Fig. 1), of approximately 430 MB in size developed using the Tkinter module, and packaged with PyInstaller for Windows. The open-source code (under the MIT license), which is included in the appendix and hosted on the GitHub repository (https://github.com/capt-clay10/RepD3D.git), is written entirely in Python. For users on UNIX systems, or those with an existing Python environment, a main.py file is available, enabling access to RepD3D's functionality via the command line. These users must install the following libraries and packages in their Python environment: ast, cfgrib, csv, dask, datetime, ecCodes, h5netcdf, h5py, math, netcdf4, numpy, os, pandas, pyproj, re, requests, seaborn, scikit-learn, scipy, statistics, sys, time, tqdm, utm, windrose and xarray.

The functionality of RepD3D can be conceptually split into two main types of modules, the representative period identification (RPI) module and extraction modules (EM). The RPI module is based on the unfiltered-reduction technique introduced by Soares et al. [18], which uses long-term wind datasets as a reference period to identify a shorter representative period that best mimics the properties of the total reference period. In the pre-compiled version, the extraction modules rely on the EasyGSH [10,11] and COSMO-REA6 [1] dataset to extract and write Delft3D-4-compatible space- and time-varying (ST) input condition files (see Fig. 5 for coverage). Together these modules help plan and prepare representative boundary conditions for a Delft3D-4 model.

## Representative period identification module

**Fig. 2:**
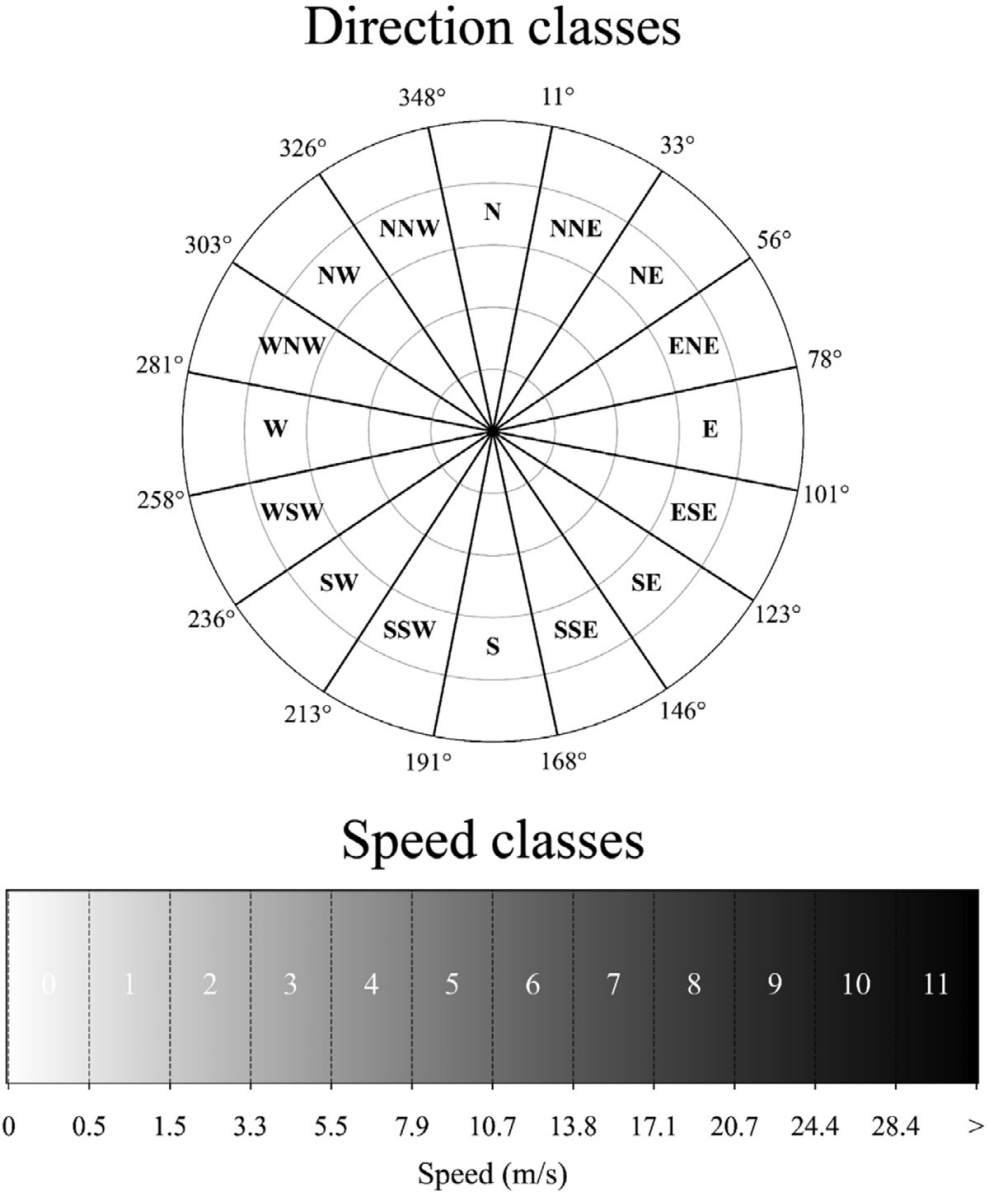
Pre-defined direction and speed classes. For further information on this classification, refer to Soares et al. [18].

The RPI module algorithm uses the summation of two correlation metrics to identify a representative period based on pre-classified direction and speed classes (Fig. 2). Metric one essentially correlates the occurrence frequency of the *relevant* direction classes with the *relevant* speed classes between a prospect period (potential period to be considered as representative) and the total reference period (total hind cast dataset) and metric two correlates the occurrence frequency of the *relevant* speed classes with the *relevant* direction classes. The *relevance* of a speed or direction class is determined by weights extracted from the total reference period for each considered direction or speed class. The summation of these two metrics gives a final Rep score, and a score approaching 0 indicates a highly representative period. This approach is statistically robust, as it evaluates full distributions of wind speed and direction rather than relying on simplistic averages. For a detailed description of the algorithm, readers are directed to the original paper [18].

**Fig. 3:**
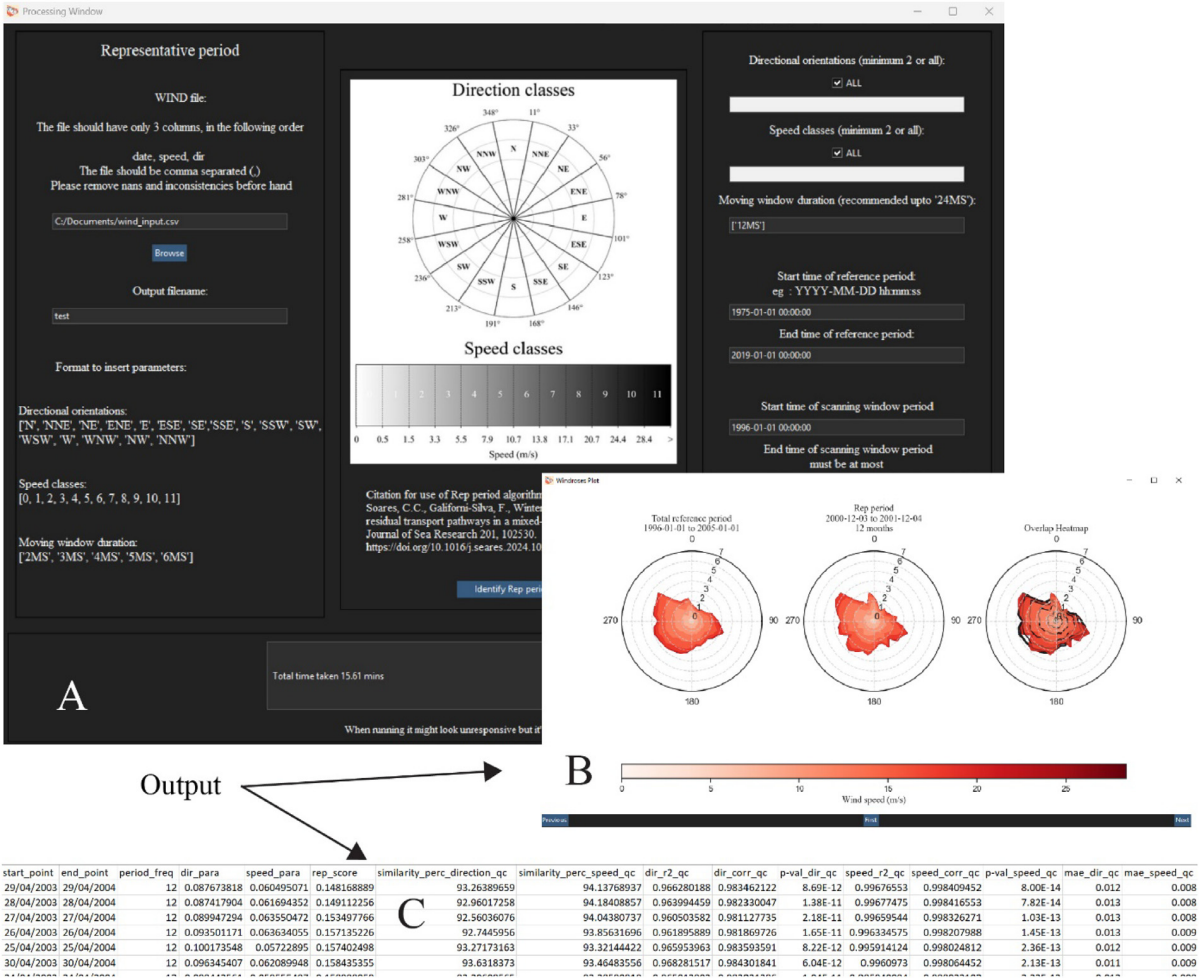
RPI module input window (A) with the associated output wind rose (B) and output file (C).

The RPI module identifies the representative period based on the user provided long-term wind data file, which represents the total reference period, along with user provided threshold values for the binning of directions and speeds (Fig. 3). The module then calculates the correlations over all prospect periods identified based on a moving window search, the size of which is defined by the user as moving-window duration (minimum 2 months). The period over which the search is conducted is referred to as the scanning window period and the limits of which are also set by the user.

(1) $$Sim\;perc=\;\frac{\sum_{i=1}^{N}\left[\left(100-\;\frac{\left|\;w_{i}-{\hat{w}}_{i}\right|\cdot 100}{w_{i}}\right)\cdot \;w_{i}\right]}{\sum_{i=1}^{N}w_{i}}\;-\;\sqrt{\sum_{i=1}^{N}{\left(w_{i}-{\hat{w}}_{i}\right)}^{2}}$$

Where $w_{i}$ = Total reference period occurrence weights, ${\hat{w}}_{i}$ = Prospect period occurrence weights and i= classes.

Along with the Rep score and the individual correlation metrics, additional error metrics, including similarity percentage (Eq. (1)), R-squared value, Pearson correlation coefficient, and mean absolute error (following Williams and Esteves [20]), are calculated as secondary quality control measures and represented by ‘*_qc’ in their names. These additional metrics evaluate the normalized occurrence frequency of directional (speed) classes during the reference periods with each prospect period separately, without accounting for the dependency of speed on direction or vice versa. The results are compiled into a .txt (Fig. 3C) file following this naming convention:

‘rep_period_(*Output_name*)_*(Scanning_window_period)_*(*Considered*_*direction_classes*)_*(Considered_speed_classes)_*(*Considered*_*moving_window_duration*).txt’.

Additionally, upon completion of the process, a new window showing the wind rose comparisons will open (Fig. 3B). Here the user can scan through the list of wind roses from the identified periods and compare it to the reference period wind rose.

## Selective-class correlation

The selective-class correlation allows users to choose specific direction and speed classes on which they wish to concentrate, in contrast to the all-class correlation. Consequently, the identified representative period will be representative only of the specified classes. This functionality enables users to identify an unfiltered special case representative period that accentuates the directions and speeds most relevant to their modeling objectives. This may be required for a coastal model of limited directional exposure to the sea.

However, it is important to recognize that this selective approach excludes other classes during the correlation process, which could be crucial to the overall modeling objective of system understanding. The exclusion of certain classes also implies that the weighting of the “selected” classes is adjusted according to the user-specified range. For instance, if a user focuses only on direction classes between East and South, the algorithm will only derive the associated weights from the total reference period from these quadrants. This may result in (for example) the SE quadrant having the highest weights, leading to a final identified period with a higher occurrence of winds in this quadrant. However, if the NW quadrant, which is excluded in this scenario, has the highest weight in the total reference period, the identified period (under the special selective scenario) may deviate from the overall representative period. However, it remains representative within the specified limits, which is the goal of using selective-class correlation over all-class correlation. In general, it is advisable to either include as many classes as possible or reduce the moving-window duration to minimize the influence of non-essential classes.

**Fig. 4:**
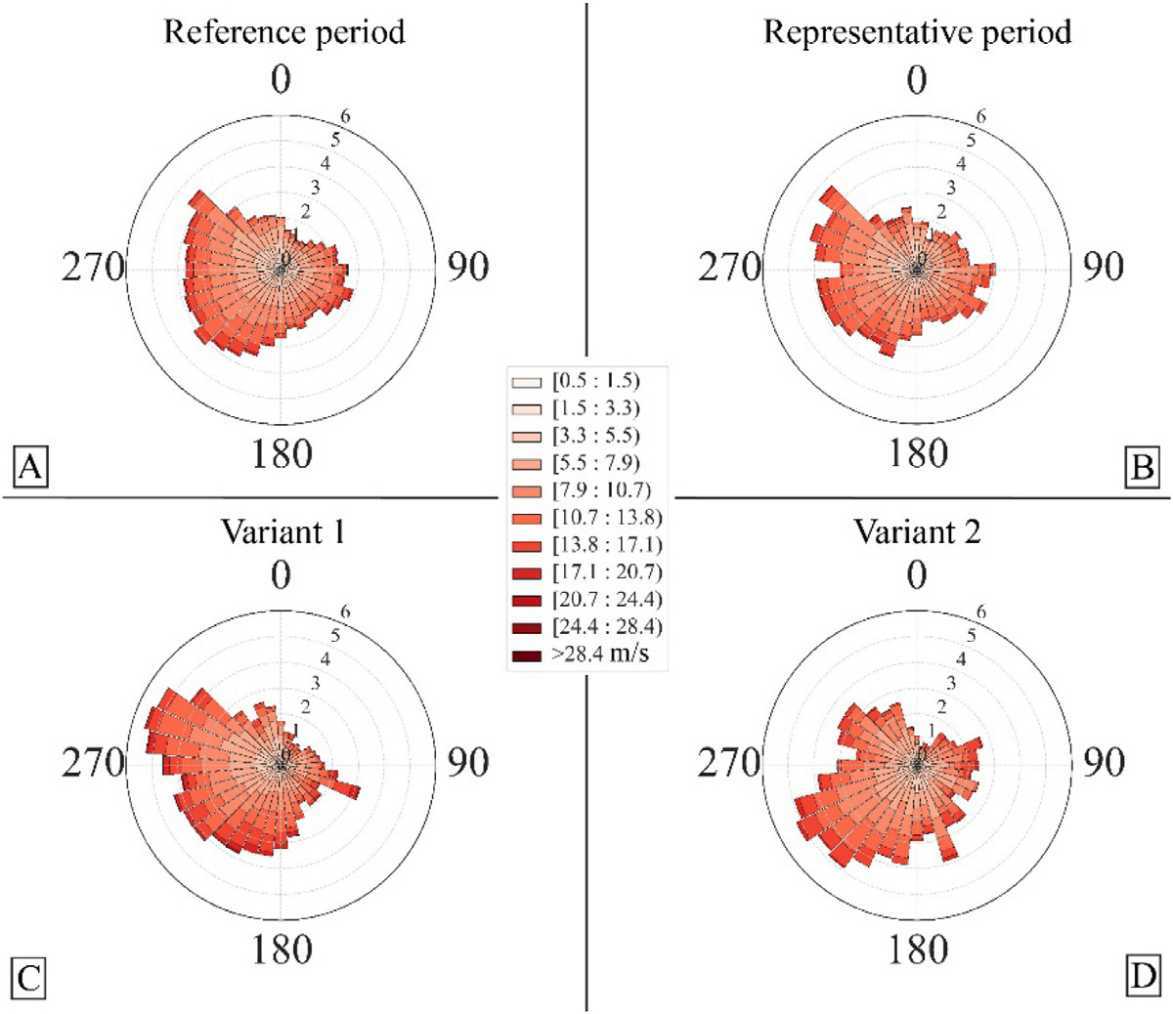
The overall reference period (A) and the resultant wind roses for periods identified by the RPI module for different user-specified settings: Representative period for all direction and speed classes (B), variant 1 (C: 12 months, speed classes 8 to 11) and variant 2 (D: 6 months, direction classes SE to SW).

Fig. 4 exemplarily illustrates the effect of selective-class correlation on the identification of representative periods, as demonstrated by wind roses generated from the identified periods. The demonstration uses wind data from the Helgoland wind station, provided by German Weather Service (Deutscher Wetterdienst - DWD). The figure presents the total reference period and the identified representative period of 12 months based on all-class correlation (Fig. 4B), alongside two variants that employ selective-class correlation. Variant 1 (Fig. 4C), with a 12-month moving-window duration, was identified by including all direction classes but restricting speed classes to those between 8 and 11. As a result, Variant 1 represents a period characterized by high-energy events, with weights adapted to wind directions typical of such events in the total reference period. Variant 2 (Fig. 4D), with a 6-month moving-window duration, was identified by including all speed classes but limiting direction classes to those within the SE to SW quadrant. Since selective correlation omits non-essential classes that could influence modeling studies, the algorithm includes quality check measures (Fig. 3C) that account for all directional and speed classes. These measures provide a general understanding of the overall characteristics of the identified period. Alternatively, this comparison can be made by first identifying the representative period based on all-class correlation and then comparing it to user-specified variants. Additionally, using the wind roses window (Fig. 3B) to review identified periods is recommended, as it offers visual support in selecting the most appropriate period.

## Extraction modules

The EM within RepD3D offer support to extract water level and wave data from the EasyGSH datasets and convert it to Delft3D-4 compatible input files as *.bct* (water level time series) and *.bcw* (wave data time series) respectively, as well as wind velocity and pressure files from COSMO-REA6 to *.amu, .amv, .amp* and *x-* and *y-wind* METEOFILES. All extracted files are currently in UTC. Implementation of these files into a model should be done following the Delft3D-4 manual [5].

**Fig. 5:**
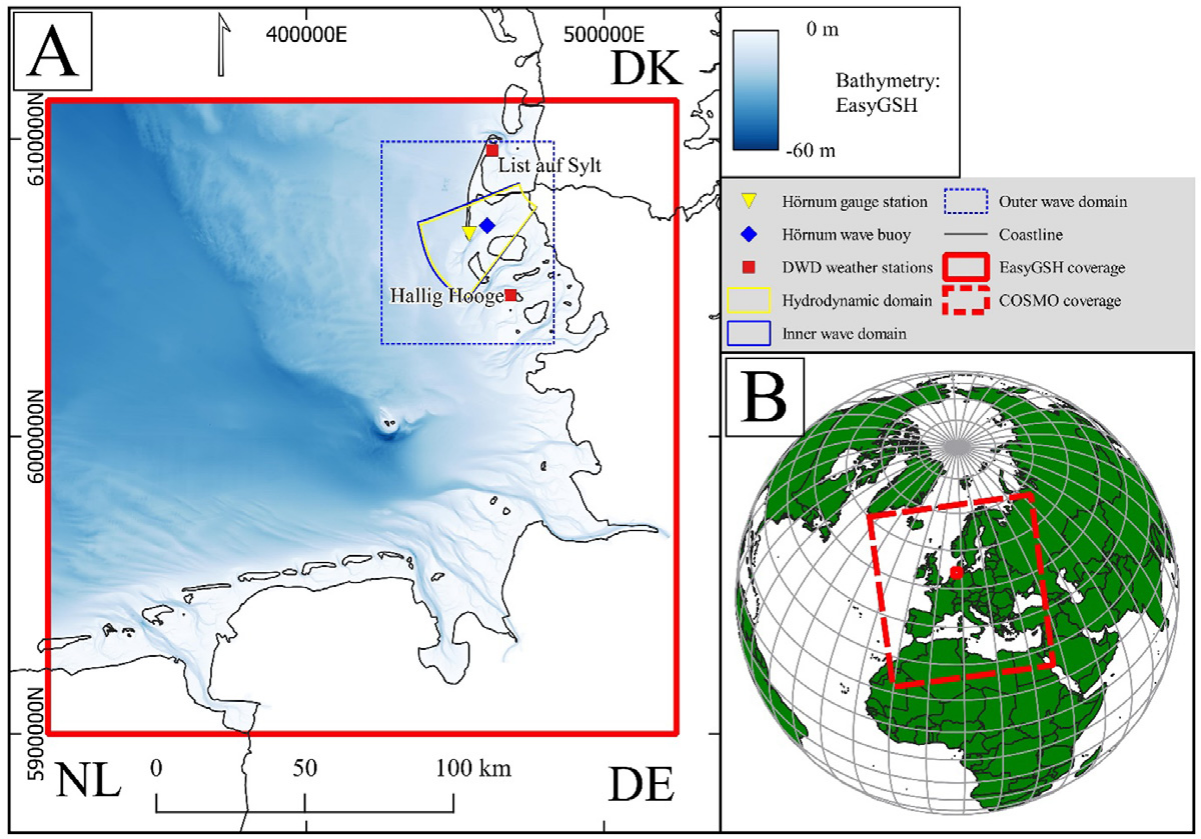
A: Overview of EasyGSH coverage and model domains / validation points of example case. B: Overview of COSMO-REA6 and EasyGSH coverage.

As an overview, the EM consists of 7 sub-modules (Table 1). Currently the modules read COSMO-REA6 for wind field data and EasyGSH for water level and wave data. This exclusivity restricts the use of the module to water level and wave conditions from the German North Sea (see EasyGSH coverage in Fig. 5A) and wind data to the European continent and the connected marginal seas (see Fig. 5B). Further enhancements are possible within the source code to adapt the algorithm to other similar datasets.

**Table 1:** Sub-modules within the extraction module and the associated generated output files.

**Sub-module**	**Input**	**Output**
Extract all files	*.mdf, .grd* (flow and wave), *.bnd* (flow and wave), *.mdw* and *.nc* (EasyGSH water level and wave)	*.bct, .bcw, .mdw*, boundary locations.csv
Bct file overlapping over years	*.mdf, .grd* (flow), *.bnd* (flow), *.nc* files (EasyGSH water level)	*.bct*, boundary locations.csv
Bcw file overlapping over years	*.mdw, .grd* (wave), *.bnd* (wave), *.nc* files (EasyGSH wave)	*.bcw, .mdw*, boundary locations utm.csv
Boundary location CSV file	*.grd* and *.bnd*	boundary locations.csv
Boundary location and mdw file	*.grd* (wave), *.bnd* (wave) and *.mdw*	*.mdw*, boundary locations.csv
Add sea level change to .bct files	*.bct*	*.bct*
Generate ST-varying wind field files - COSMO	*.grb* (PS, U and V) and *.mat* (DelftD3D-4 grid, exported as .mat)	.*amu, .amv, .amp, xwind.wnd, ywind.wnd*

In the EM, the algorithm extracts the A and B endpoints of each open boundary provided in the user-specified open boundary file (*.bnd*) (Table 2). It converts them into real-world coordinates from *m* and *n* indices (Cartesian to Spherical if needed) using information from the provided grid file (*.grd)*. This initial step can also be performed independently using the sub-module *Boundary location CSV file*. The A and B coordinates are then used as indices to extract data from the EasyGSH variables. The algorithm implements a bilinear interpolation technique (Eq. (2)) to extract information for each boundary endpoint, this offers more precision if the user-provided boundaries lie in an area with high variability, however, it increases the run time of the algorithm. Four bounding points, nearest to the target coordinates are identified and data ($P_{i})$ is extracted at each of these locations.

(2) $$Bli=\;\frac{\sum_{i=1}^{4}P_{i}\cdot \;w_{i}}{\sum_{i=1}^{4}w_{i}}$$

where $P_{i\ldots 4}$ represents the extracted data points nearest to the target coordinates and $w_{i..4}$ the associated weights based on their relative distances from the target coordinates.

**Table 2:** Important warnings and recommendations related to the usage of RepD3D.

Input file	Where to prepare	Warnings and recommendations
*.grd* (flow and wave)	Delft3D-4 - Flow module or outside	Please make your flow and wave grids as desired using the RGFGRID, Cartesian space or Spherical.
*.bnd* (flow)	Delft3D-4 - Flow module or outside	Select the type of open boundary as **Water level** and forcing type as **Time-series** Name the boundaries **with** an underscore and follow that with a number, eg North_1, **do not use North_01**
*.bnd* (wave)	Delft3D-4 - Flow module or outside	Use the Delft3D-4 flow module to make boundaries on your wave grid, to achieve a space-varying effect make several boundaries Name the boundaries **without** an underscore, eg North1, **do not use North01** It is recommended to use a larger rectangular outer wave grid to which you prescribe the boundaries and then nest (at least) a higher resolution wave grid (rectilinear or curvilinear) within it that eventually communicates with your flow grid. *Note all wave grids should be bigger than flow grids for best results.
*.mdf* (flow)	Delft3D-4 - Flow module or outside	The reference date and simulation start and stop time should be inserted carefully.
*.mdw* (wave)	Delft3D-4 - Wave module or outside	Make sure at least one interim boundary is set up for the algorithm to find the *Boundaries* block in the *.mdw* file. This will be overwritten by your created boundaries from the *.bnd* (wave) file.
*.nc* waterlevel file (EasyGSH)	EasyGSH	Download the *.nc* file to the main path from https://mdi-de.baw.de/easygsh/Easy_Viewer_syn.html#home File is found under *synoptische Simulation, UnTRIM2, 1000m Raster* as *Wasserstand 2D* Do not change the name of the *.nc* files
*.nc* wave file (EasyGSH)	EasyGSH	Download the .nc file to the main path from https://mdi-de.baw.de/easygsh/Easy_Viewer_syn.html#home File is found under *synoptische Simulation, UnTRIM2, 1000m Raster* as *Seegang 2D* Do not change the name of the *.nc* files
WIND file	DWD or outside	The file should be cleaned of nan values The file should only have 3 columns in the following order: **date, speed** and **direction**.
COSMO wind files	https://opendata.dwd.de/climate_environment/REA/COSMO_REA6/hourly/2D/	In the main path make two folders called PS and UV. In the folder PS place, all the pressure files (PS) for the months you're interested In the UV folder place, all the U_10 M and V_10 M files for the months you're interested in. Unzip the files such that the final format is now .*grib*. and delete the zip files. It is important to note that the reference time for the COSMO files should be the reference date of the model simulation, however make sure there is a time gap between the start of the COSMO data and the reference time. So, if your reference time is 01.01.2024 then download COSMO files starting from 01.02.2024.

The following variables are extracted from the EasyGSH data set (all times in UTC), for water level data, it extracts *Mesh2_face_Wasserstand_2d* which represents sea surface height or water level. For wave data, it extracts *Mesh2_face_signifikante_Wellenhoehe_2d* which represents significant wave height, *Mesh2_face_Peak_Wellenperiode_2d* which represents peak wave period, *Mesh2_face_Richtungsaufweitung_der_Wellen_2d* which represents directional spreading and *Mesh2_face_Wellenrichtungsvektor_x_2d* and *Mesh2_face_Wellenrichtungsvektor_y_2d* which represent the two components of wave direction. These components are then processed for each quadrant to accurately calculate the directional angle (Nautical convention). Finally, the calculated angle is adjusted to convert the wave orientation from the 'waves towards' direction to the 'waves from' direction.

Sub-modules *Bct file overlapping over years* and *Bcw file overlapping over years* allow the user to extract data for model simulations spanning more than one year. A supplementary sub-module called *Add sea level change to .bct files* provides the opportunity to induce gradual or constant sea level rise to the extracted *.bct* files. For inducing gradual SLR it requires a maximum SLR height in meters then it extrapolates an array from zero to the provided max in the same resolution as the time step in the *.bct* file and simply adds it over the water levels in the *.bct* file.

The COSMO-REA6 wind fields are extracted and interpolated onto an equidistant grid with a resolution of 6 × 6 km, the time is in UTC. The grid has to be provided by the user in *.mat (v7)* format, this can be generated easily using the Quick plot function in Delft3D-4. COSMO-REA6 data is provided on a rotated pole, which is corrected within the EM before extraction. The user only needs to follow the folder structure specification (Table 2). The output files generated include wind components (U and V) and stored in files *.amu* and *.amv*, air pressure component stored in the file *.amp*, formatted for use in Delft3D-4 Flow module and METEO files for the wave module stored in the files *.wnd*.

## Usage guidelines

RepD3D is relatively user friendly with several hints and instructions mentioned in the user interface for smooth operations. A detailed list of guidelines is presented in Table 2 to support in preparation before using the GUI.

## Method validation

Soares et al. [18] employed the RPI module to identify a representative period using the all-class correlation method and extraction modules to obtain relevant input conditions for their North Frisian Wadden Sea model. Validation in their study demonstrated that the generated input conditions performed effectively. This section presents a simplified use case scenario illustrating how RepD3D can be applied in modeling efforts, with example files provided as tutorial resources on the GitHub repository.

**Fig. 6:**
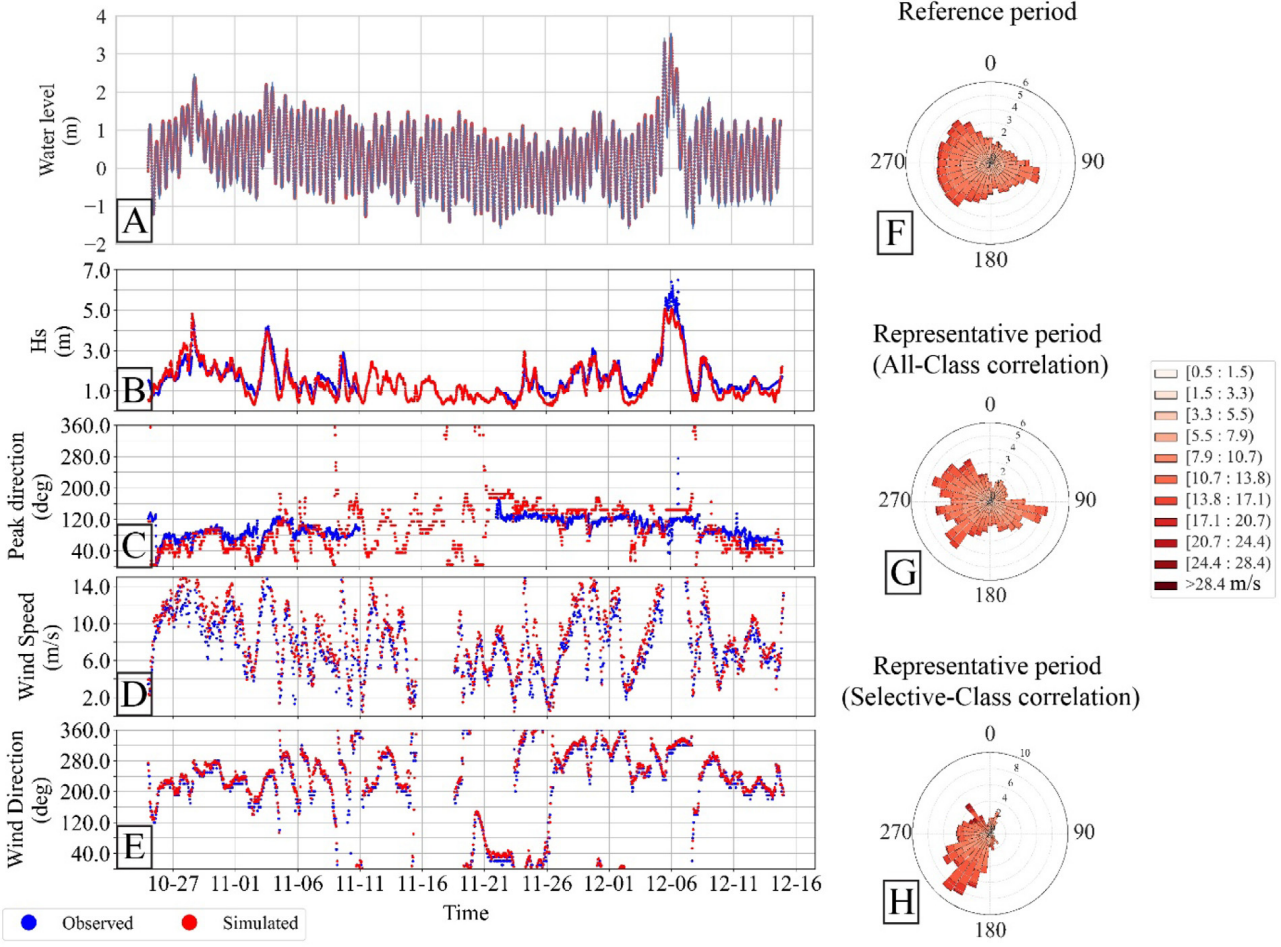
Application of the EM and RPI modules for an example scenario. Left panels A-E compare observed (blue) and simulated (red) water level, wave and wind parameters. Right panels show wind roses for the reference period (F), representative period for all direction and speed classes (G) and for the user-specified classes (H) used in the example.

For this exemplary case study, we focus on the back-barrier basin Hörnumbecken (situated between the Island of Sylt and Amrum, model domain as depicted in Fig. 5A), where the objective is to analyze the impact of strong winds from the southwest quadrant on the regional hydrodynamics. The goal is to conduct a non-synthetic, unfiltered-reduced but representative simulation using the RPI module. Wind data for the identification of the (selective-class) representative period was sourced from the List weather station on Sylt, provided by DWD. While it is generally advised to use data from an offshore wind station, the data from this station was deemed sufficient for the current scenario. Using the selective-class correlation method, we chose wind directions from the S, SSW, SW, and WSW quadrants, along with wind speeds in classes 8, 9, 10, and 11. To minimize the influence of less relevant classes, the lowest moving-window duration of two months was selected, with the scanning window limited to overlap with EasyGSH and COSMO datasets. Fig. 6H illustrates the representative period identified based on the reference period (1975-01-01 to 2020-01-01), spanning from October 26, 2013, to December 24, 2013, aligning with the modeling objective.

A Delft3D-4 based simplified low resolution curvilinear structured grid was generated for the Hörnumbecken basin, with the highest resolution in the channel and back-barrier areas, and reduced resolution toward the open sea. Two wave grids were developed: a low-resolution outer rectilinear wave grid and an inner wave grid matching the resolution of the flow grid. Space- and time-varying meteorological files from the COSMO model were used for the wave (SWAN) module, while the flow module incorporated the *.bct* file from EasyGSH for water levels. The results, presented in Fig. 6 (A-E), demonstrate the application's effectiveness. The figure Fig. 6 (F-H) shows the wind rose comparison between the reference period, representative period identified under all-class correlation and the selective-class correlation method used for this scenario. It also qualitatively shows the water level, wave and wind data comparison between measured and modeled (extracted using EM and run with Delft3D-4), the locations of the measured stations can be seen in Fig. 5A.

## Limitations

While RepD3D serves as a valuable companion tool to modelers, specially to Delft3D-4 users, there are limitations and areas for improvement that should be addressed in future iterations:

• The current RPI module excels in providing statistically representative conditions; however, it does not capture the chronology of events, such as storm sequences. This limitation has been highlighted in studies like Eichentopf et al. [7], which emphasize the importance of event sequencing on hydrodynamic and morphodynamic outcomes. Incorporating event chronology would significantly enhance the module's realism and broaden its application scope.
• At present, the Extraction Module (EM) is restricted to the German North Sea, using water level and wave data from the EasyGSH dataset. While this dataset provides valuable information, expanding the module's spatial coverage to include the TrilaWatt dataset (https://trilawatt.eu/), the successor to EasyGSH, would extend its applicability to regions across the Netherlands, Germany, and Denmark. Similarly, integration with additional datasets (Copernicus, ERA5 and more) could allow for broader coverage.
• The current toolbox lacks functionalities to introduce gradual or constant variations to wave and wind data in boundary condition files, as well as the ability to create artificial boundary conditions for synthetic simulations. Adding these capabilities would improve flexibility for users conducting specialized modeling experiments.
• The graphical user interface (GUI) of RepD3D could be optimized to handle computationally intensive tasks more efficiently. Implementing multithreading or similar performance-enhancing techniques would streamline the user experience, especially for large datasets or complex simulations.
• To stay aligned with the evolving needs of users, continuous updates and community involvement are essential. Users are encouraged to monitor the project repository (https://github.com/capt-clay10/RepD3D.git) for ongoing improvements and contribute suggestions or enhancements to the tool.

## CRediT author statement

**Clayton Soares:** Conceptualization, Methodology, Software, Validation, Formal analysis, Writing-Original Draft, Visualization. **Arne Knies:** Software, Validation, Writing-Review & Editing, Visualization. **Christian Winter:** Writing-Review & Editing, Supervision, Funding acquisition.

## Declaration of competing interest

The authors declare that they have no known competing financial interests or personal relationships that could have appeared to influence the work reported in this paper.

## Data Availability

All source code is linked in the manuscript.

## Appendix Supplementary materials

Image, application 1

Image, image 2
