# Supplementary figures and images for: RepD3D: A tool for representative period identification and associated boundary condition extraction
Source: MethodsX. 2024 Dec 13;14:103109. doi: 10.1016/j.mex.2024.103109 (PMC12255363; doi:10.1016/j.mex.2024.103109)

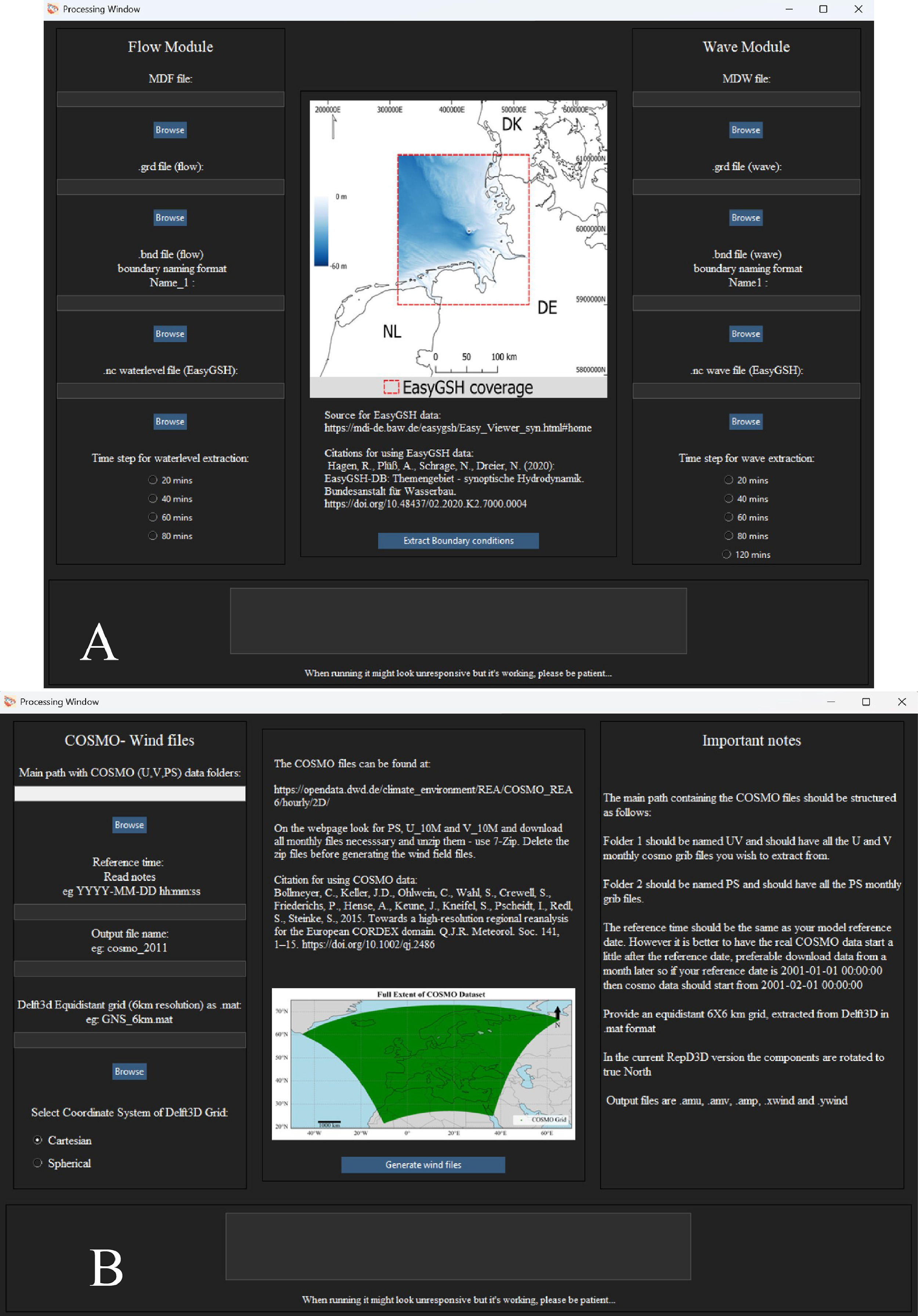

Supplement: Supplementary file 2 [file mmc2.jpg]
